# Complementary recognition of the receptor-binding site of highly pathogenic H5N1 influenza viruses by two human neutralizing antibodies

**DOI:** 10.1074/jbc.RA118.004604

**Published:** 2018-08-28

**Authors:** Yanan Zuo, Pengfei Wang, Jianfeng Sun, Shichun Guo, Guiqin Wang, Teng Zuo, Shilong Fan, Paul Zhou, Mifang Liang, Xuanling Shi, Xinquan Wang, Linqi Zhang

**Affiliations:** From the ‡ Comprehensive AIDS Research Center, Collaborative Innovation Center for Diagnosis and Treatment of Infectious Diseases, Department of Basic Medical Sciences, School of Medicine, Tsinghua University, Beijing 100084,; the § Ministry of Education Key Laboratory of Protein Science, Beijing Advanced Innovation Center for Structural Biology, Collaborative Innovation Center for Biotherapy, School of Life Sciences, Tsinghua University, Beijing 100084,; the ¶ Department of Computer Science, Tsinghua University, Beijing 100084,; the ** State Key Laboratory for Infectious Disease Control and Prevention, National Institute for Viral Disease Control and Prevention, Chinese Center for Disease Control and Prevention, Beijing 100084, and; the ‖ Unit of Anti-Viral Immunity and Genetic Therapy, Key Laboratory of Molecular Virology and Immunology, Institute Pasteur of Shanghai, Chinese Academy of Sciences, Shanghai 200031, China

**Keywords:** influenza, influenza virus, monoclonal antibody, structural biology, X-ray crystallography, vaccine development, virology, mutagenesis, infectious disease, antigenic variation, escape mutants, H5N1, HPAI, neutralizing antibodies, receptor-binding site, antiviral vaccine

## Abstract

The highly pathogenic avian influenza virus H5N1 is a major threat to global public health and therefore a high-priority target of current vaccine development. The receptor-binding site (RBS) on the globular head of hemagglutinin (HA) in the viral envelope is one of the major target sites for antibody recognition against H5N1 and other influenza viruses. Here, we report the identification and characterization of a pair of human RBS–specific antibodies, designated FLD21.140 and AVFluIgG03, that are mutually complementary in their neutralizing activities against a diverse panel of H5N1 viruses. Crystallographic analysis and site-directed mutagenesis revealed that the two antibodies share a similar RBS-binding mode, and their individual specificities are governed by residues at positions 133a, 144, and 145. Specifically, FLD21.140 preferred Leu-133a/Lys-144/Ser-145, whereas AVFluIgG03 favored Ser-133a/Thr-144/Pro-145 residue triplets, both of which perfectly matched the most prevalent residues in viruses from epidemic-originating regions. Of note, according to an analysis of 3758 H5 HA sequences available in the Influenza Virus Database at the National Center for Biotechnology Information, the residues Leu-133a/Ser-133a and Ser-145/Pro-145 constituted more than 87.6 and 99.3% of all residues at these two positions, respectively. Taken together, our results provide a structural understanding for the neutralizing complementarity of these two antibodies and improve our understanding of the RBS-specific antibody response against H5N1 infection in humans.

## Introduction

Since its first discovery in a sick goose in Guangdong, China, during the summer of 1996, highly pathogenic avian influenza virus (HPAI)^4^ H5N1 was attested to be a major threat to global public health. With its unusual pathogenicity, HPAI H5N1 has caused frequent disease outbreaks in domestic poultry farms across the world and has resulted in millions of deaths among chickens, ducks, and geese (1–5). The HPAI H5N1 virus has also managed to cross the species barrier to infect humans, causing human disease with an exceedingly high mortality rate of more than 52% (http://www.who.int/influenza/human_animal_interface/H5N1_cumulative_table_archives/en/, accessed March 2, 2018)^5^ (6, 7). Mutations in the viral envelope and internal genes enable its entry and replication in a broader range of cell types as well as escape from antiviral immunity (8–14). Fortunately, the current HPAI H5N1 strains have rather inefficient transmission among humans and other mammals. However, recent “gain-of-function” studies showed that only a few mutations are sufficient for the virus to become transmissible by airborne droplets in ferrets and guinea pigs (8–10), raising serious concerns about its pandemic potential in the near future. There is an urgent need for the global community to develop effective diagnostics, vaccines, and therapeutics to prevent its further spread into the human population.

HPAI H5N1 strains isolated from humans worldwide represent a divergent and evolving cluster of quasi-species that can be broadly classified into 10 clades (designated 0 through 9) and a large number of subclades (15–17). China, Vietnam, Egypt, Cambodia, Indonesia, and Thailand are among the countries with the highest rates of human H5N1 infection and harbor the highest complexity of H5N1's genetic diversity (17–19). The genetic diversity is primarily manifested in the viral envelope hemagglutinin (HA), which facilities viral entry into the target cells and evasion of recognition by the human immune response. As an integral membrane glycoprotein, HA forms homotrimeric spikes at the surface of the virion (20). Each monomer is initially synthesized as a full-length precursor HA0 and then cleaved into HA1 and HA2 subunits by host cell proteases (20). One unique feature of HPAI H5N1 is the presence of polybasic residues at the cleavage site allowing recognition by ubiquitous proteases, and thereby promoting replication in diverse cell types (21–23). Structurally, the HA1 subunit resembles a globular head, whereas the HA2 subunit adopts a hairpin form composed of two antiparallel α-helixes (24–27). The HA1 subunit contains a receptor-binding site (RBS) for binding of the virion to the cellular receptor sialic acid, whereas the HA2 subunit mediates the subsequent fusion between the viral envelope and the host's cellular endosomal membrane (20). The RBS consists of a number of conserved hydrophobic and aromatic residues at its base and three conserved elements, a 130-loop, 190-helix, and 220-loop at its edge (28–31). Despite the relatively conserved sequence and overall structure, the RBS contains critical residues that strongly affect the receptor-binding preference across different species and alter the interaction mode among different subtypes. In particular, the RBS of HPAI H5N1 has the potential to evolve and to acquire and improve an affinity for the human α2,6-linkage over avian α2,3-linkage in the surface receptors, and therefore it broadens its tropism to a greater variety of cell types in humans and other species alike (8–10, 32).

In influenza infection and vaccination, neutralizing antibody responses against HA play a critical role in protecting humans from infection and disease progression (33–37). Through systematic analysis of H5-specific neutralizing mAbs with available clear structural information, we have previously defined four major vulnerable sites (VS1–4) on the globular head of H5N1 HA (34). Since then, we continue to characterize additional H5-specific human antibodies with a hope to further improve and optimize our initial structural definition of the globular head of HA. During this process, we have come across a pair of antibodies, designated FLD21.140 and AVFluIgG03, that are unique in their mutual complementarity of binding and their neutralizing activities against a panel of diverse H5N1 strains. AVFluIgG03 was derived from the panning of an antibody phage library from the Chinese patient AH06 who recovered from HPAI H5N1 infection in 2006 (38). FLD21.140 was derived from the Epstein-Barr virus-immortalized memory B cells of the convalescent Vietnamese patient CL115 who was infected with H5N1 in 2005 (39). Based on our previous structural analysis, AVFluIgG03 appears to recognize the RBS on the globular head of A/Anhui/1/2005 HA (34). Here, we report a structural and functional comparison between FLD21.140 and AVFluIgG03. Our results showed that the two antibodies shared a similar mode of binding to the RBS, and their respective specificities were determined by only three residues at positions 133a, 144, and 145. FLD21.140 favored the Leu-133a/Lys-144/Ser-145 combination, whereas AVFluIgG03 preferred Ser-133a/Thr-144/Pro-145, both of which matched perfectly with the dominant residues of circulating viruses in the epidemic regions where they were isolated. Despite significant fluctuation over time, the residues Leu-133/Ser-133 and Ser-145/Pro-145 account for over 87.6 and 99.3% of all residues at these two positions, respectively, based on the analysis of 3758 H5 HA sequences currently available in the Influenza Virus Database at the National Center for Biotechnology Information. All in all, our data explain the unique complementarity of these two antibodies from the perspective of structural analysis and should aid a better understanding of the RBS-specific antibody response against human H5N1 infection.

## Results

### FLD21.140 and AVFluIgG03 neutralize distinct but complementary subsets of H5N1 influenza viruses

Our own group and others have previously characterized a group of human monoclonal antibodies isolated from the convalescent patients infected by the highly pathogenic H5N1 virus (38–41). While testing their neutralizing activities against a panel of diverse H5N1 strains, we identified a pair of antibodies, designated FLD21.140 and AVFluIgG03, that are unique in their mutual complementarity in binding and neutralizing activities. Both antibodies demonstrated a high degree of similarity to their respective germline genes (>95%) suggesting they are derived from B cells without going through an extended period of maturation *in vivo* (Table S1). Of the 18 diverse H5N1 strains, those sensitive to FLD21.140 were less sensitive or virtually resistant to AVFluIgG03 and vice versa. For example, FLD21.140 strongly neutralized strains from clades 0, 1, 2.2.1, 2.3.2.1, 2.4, 2.5, 3, 4, and 8, whereas AVFluIgG03 only neutralized those from the remaining clades 2.1.3.2, 2.3.4, 5, 6, 7, 7.1, and 9 (Table 1). FLD21.140 also exhibited a much broader neutralizing activity than AVFluIgG03. Among the 18 diverse strains tested, FLD21.140 was able to neutralize 88.89%, whereas AVFluIgG03 neutralized 61.11% of all strains (Table 1). Furthermore, binding affinities for the globular head proteins mirrored the antibody-neutralizing activities. Among the five representative strains, FLD21.140 demonstrated strong binding to A/Thailand/1(KAN-1)/2004 and A/Vietnam/1203/2004 from clade 1, as well as A/Turkey/65596/2006 from clade 2.2.1, but weak binding to A/Anhui/1/2005 and A/Beijing/01/2003 from clade 2.3.4 and clade 7, respectively (Table 2 and Fig. S1). By contrast, AVFluIgG03 strongly bound the HA head of strains from clades 2.3.4 and 7 but weakly to those from clades 1 and 2.2.1 (Table 2 and Fig. S1). Such dichotomy in their neutralizing and binding specificities was likely due to the distinct antibody response to the different infecting viruses in patients from whom the two antibodies were initially isolated. Indeed, FLD21.140 was isolated from the Vietnamese patient CL115, who was infected with the clade 1 strain A/Vietnam/CL115/2005, whereas AVFluIgG03 was from the Chinese patient AH06 infected with the clade 2.3.4 strain A/Anhui/1/2005 (38, 39).

**Table 1:** **Neutralization activity of antibodies FLD21.140 and AVFluIgG03 against H5N1 pseudoviruses belonging to different clades**

Strain	Clade	IC_50_*^a^* (μg/ml)	
		FLD21.140	AVFluIgG03
A/Hong Kong/156/1997	0	0.281	>50.000
A/Thailand/1(KAN-1)/2004	1	0.008	>50.000
A/Vietnam/1203/2004	1	0.001	>50.000
A/Indonesia/5/2005	2.1.3.2	13.600	1.132
A/Turkey/65596/2006	2.2.1	0.001	1.861
A/Common Magpie/Hong Kong/5052/2007	2.3.2.1	0.050	>50.000
A/Anhui/1/2005	2.3.4	2.109	0.002
A/Chicken/Guangxi/12/2004	2.4	0.076	>50.000
A/Chicken/Korea/ES/2003	2.5	0.034	16.667
A/Silk Chicken/Hong Kong/SF289/2001	3	0.003	0.115
A/Goose/Guiyang/337/2006	4	0.006	>50.000
A/Duck/Guangxi/1378/2004	5	4.097	0.008
A/Blackbird/Hunan/1/2004	6	>50.000	0.004
A/Duck/Hubei/wg/2002	6	16.667	0.142
A/Beijing/01/2003	7	0.261	0.003
A/Chicken/Vietnam/NCVD-016/2008	7.1	>50.000	0.503
A/Chicken/Henan/16/2004	8	0.008	>50.000
A/Goose/Shantou/162/2005	9	2.508	0.027

*^a^* IC_50_ indicates the half-maximal inhibition concentration.

**Table 2:** **Binding affinity of antibodies FLD21.140 and AVFluIgG03 to different HA head (Asp-55–Glu-271) proteins of H5N1 virus**

HA head (Asp-55–Glu-271)	Clade	FLD21.140		AVFluIgG03	
		EC_50_*^a^*	*K_D_**^b^*	EC_50_	*K_D_*
		μ*g/ml*	*m*	μ*g/ml*	*m*
A/Thailand/1(KAN-1)/2004	1	0.022	1.004E-9	>50.000	No binding
A/Vietnam/1203/2004	1	0.008	7.017E-10	>50.000	No binding
A/Turkey/65596/2006	2.2.1	0.075	NT*^c^*	2.207	NT
A/Anhui/1/2005	2.3.4	>50.000	1.214E-5	0.057	1.262E-8
A/Beijing/01/2003	7	34.650	1.866E-6	0.055	2.500E-8

*^a^* EC_50_ indicates the half-maximal effective concentration.

*^b^ K_D_* (m) indicates the binding affinity of the two Fab fragments with four HA globular heads (Asp-55–Glu-271) as measured by SPR.

*^c^* NT means not tested.

### Crystal structure of FLD21.140 bound to the globular head of HA

**Figure 1.**
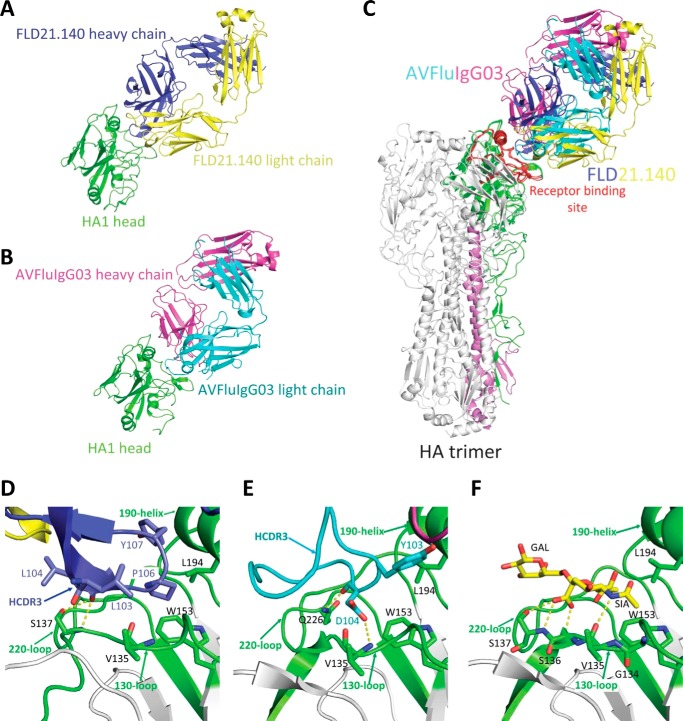
**Crystal structures of the globular head complexed with the Fab of FLD21.140, AVFluIgG03, and an α2,3-sialoglycan receptor analog.** *A,* crystal structure of the globular head and FLD21.140 Fab complex. The head is shown in *green*, the antibody heavy chain in *blue,* and light chain in *yellow. B,* crystal structure of the globular head and AVFluIgG03 Fab complex. The head is shown in *green*, the antibody heavy chain in *cyan,* and the light chain in *magenta. C,* globular head and FLD21.140 Fab and AVFluIgG03 Fab complexes are superimposed onto the A/Anhui/1/05 HA trimer. HA1 is shown in *green*, the receptor-binding site (*RBS*) in *red,* and HA2 in *violet* on one of three monomers. *D–F,* enlarged focused view of interactions between the globular head and FLD21.140 HCDR3 (*D*), AVFluIgG03 HCDR3 (*E*), and the α2,3-sialoglycan receptor analog (*F*) (PDB code 4BGY), with selected residues shown as *sticks*. The head is shown in *gray*, and the 130-loop, 190-helix, and 220-loop of RBS are in *green*.

**Figure 2.**
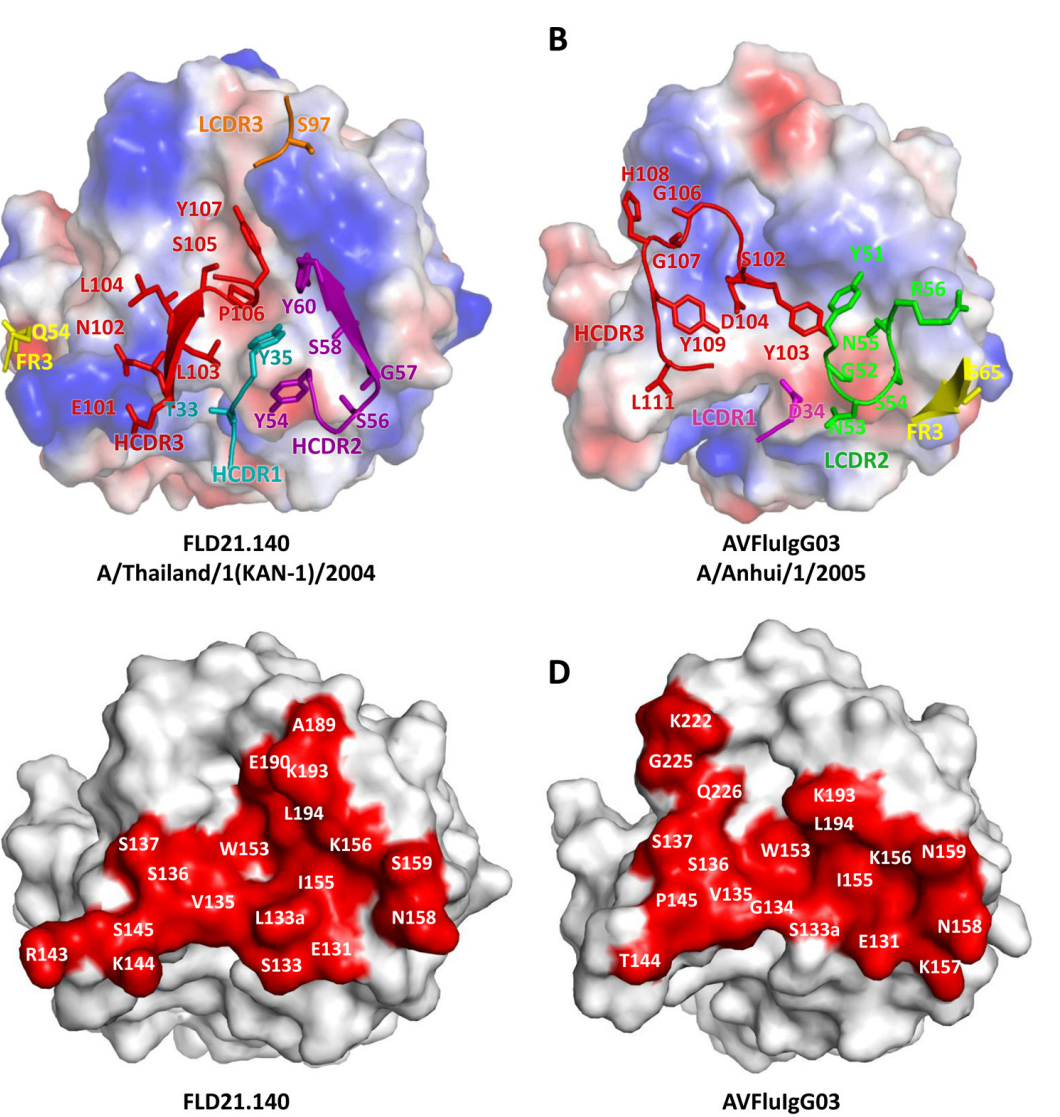
**Paratopes of FLD21.140 and AVFluIgG03 and epitopes on the globular head.** *A* and *B,* electrostatic potential surface of the globular head is illustrated (*red*, negative; *blue*, positive; *white*, neutral), together with the contacting residues in the complementarity determining regions of FLD21.140 (*A*) and AVFluIgG03 (*B*). *C* and *D,* footprints of FLD21.140 (*C*) and AVFluIgG03 (*D*) are colored in *red* on the surface of the globular head of A/Thailand/1(KAN-1)/2004 and A/Anhui/1/2005, respectively. Residues within the footprint are labeled.

To study the structural basis for the abovementioned dichotomy between FLD21.140 and AVFluIgG03, we determined the crystal structure of FLD21.140 Fab bound to the globular head of A/Thailand/1(KAN-1)/2004 at a resolution of 2.33 Å (Table S2), and we compared it with the structure of AVFluIgG03 we published previously (34). As shown in Fig. 1, the overall structure of FLD21.140 Fab bound to the globular head of A/Thailand/1(KAN-1)/2004 shared a high degree of similarity with that of AVFluIgG03 Fab bound to A/Anhui/1/2005 (34). Superimposing the two complexes onto the A/Anhui/1/2005 HA trimer demonstrated that the epitope of FLD21.140 on an HA monomer overlapped with that of AVFluIgG03, largely involving the RBS near the membrane-distal end of the trimeric spike (Fig. 1*C*). FLD21.140 covered a total surface of 753 Å^2^ on the globular head, of which 654 Å^2^ were by the heavy chain and 112 Å^2^ were by the light chain. Similarly to several published antibodies such as AVFluIgG03 (34, 42–49), recognition by FLD21.140 is largely mediated by the HCDR3 region through direct insertion into the shallow receptor-binding pocket framed by the 130-loop, 190-helix, and 220-loop (Fig. 1, *D* and *E*). Specifically, the hydrophobic residues Leu-103 and Leu-104 of FLD21.140, respectively, interacted with Val-135 and Ser-137 in the 130-loop of RBS through hydrophobic interactions and hydrogen bonds. The backbone carbonyl oxygen of Leu-104 in FLD21.140 formed two hydrogen bonds with Ser-137, one through the side chain hydroxyl oxygen and the other through the backbone amide nitrogen. Another hydrophobic residue, Pro-106, and the aromatic residue Tyr-107 of FLD21.140 interacted with HA residues Trp-153 and Leu-104 through hydrophobic and van der Waals interactions, respectively. Furthermore, residue Pro-106 inserted its side chain into the hydrophobic receptor-binding pocket surrounded by residues Trp-153 and Leu-104 in the 190-helix of RBS (Fig. 1*D* and Table S3). Both residues were highly conserved, being present in 99.9 and 95.7% of 3758 influenza H5 HA sequences currently available in the Influenza Virus Database at the National Center for Biotechnology Information, respectively (Table S4). As shown previously (50), the contact residues Val-135, Ser-137, Trp-153, and Leu-104 were also involved in the interaction with the sialoglycan receptor analog, suggesting that FLD21.140 and AVFluIgG03 exert their neutralizing activities through direct competition with the sialic acid receptor (Fig. 1, *D–F*, and Table S3). Furthermore, analogously to AVFluIgG03, FLD21.140 also used the HCDR1, HCDR2, HCDR3, LCDR3, and FR3 regions to interact with additional residues within and surrounding the RBS (Fig. 2, *A* and *B*). For example, the HCDR1 loop contacted residues Ser-133 and Leu-133a in the 130-loop. The HCDR2 loop contacted residues Glu-131, Ser-133, and Leu-133a in the 130-loop, Lys-156, Asn-158, and Ser-159 in the 150-loop, and Lys-193 in the 190-helix. In addition to the specific interactions mentioned above, the HCDR3 loop also contacted residues Leu-133a and Ser-136 in the 130-loop, Lys-144 and Ser-145 in the 140-loop, Ile-155 in the 150-loop, as well as Glu-190 and Lys-193 in the 190-helix (Fig. 2, *C* and *D*). The complex was further stabilized by these contacts.

### Critical residues determining the specificity and complementarity of FLD21.140 and AVFluIgG03

**Figure 3.**
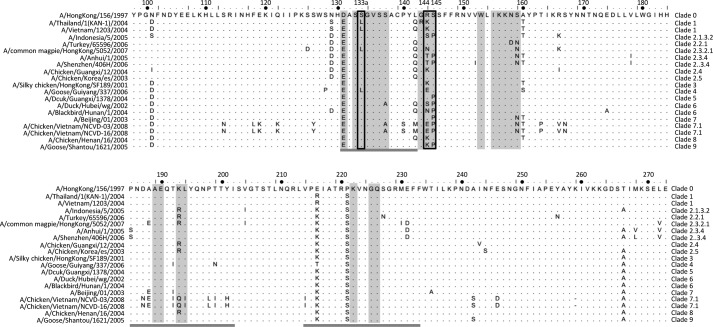
**HA amino acid sequence alignment of the 18 diverse H5N1 strains.** All epitope residues (distance ≤4 Å) recognized by FLD21.140 and AVFluIgG03 are shown in *gray*. The receptor-binding site (130-loop, 190-helix, and 220-loop) is *underlined* in *dark gray*. The key residues that determine antibody specificity, 133a, 144, and 145, are highlighted with a *black box*.

To identify critical residues that differentiate the specificity and complementarity of FLD21.140 and AVFluIgG03, we generated a total of 52 single, 6 double, and 2 triple mutant HA envelope sequences on the backbone of clade 1 A/Vietnam/1203/2004 and clade 2.3.4 A/Anhui/1/2005 (Table 3). The two strains were selected to match that in the infected individuals from whom the antibodies FLD21.140 and AVFluIgG03 were initially isolated. This way, specific and cross-reactive activity of each antibody could be readily studied. The residues selected for mutation were the contacting residues within and surrounding the RBS identified through the structural analysis (Table S3) and their prevalence among 18 diverse H5N1 strains initially used for characterization of the two antibodies (Fig. 3). Pseudoviruses bearing these mutant HA envelope sequences were then generated and subjected to sensitivity analysis by FLD21.140 and AVFluIgG03 neutralization. As shown in Table 3, different mutants had a different impact on neutralization sensitivity. Of the 25 single mutants of the A/Vietnam/1203/2004 envelope, 9 conferred a more than 10-fold increase of resistance to FLD21.140 compared with the original WT envelope. In particular, the A/Vietnam/1203/2004 L133aS mutation, corresponding to an amino acid exchange with A/Anhui/1/2005, had opposite effects on FLD21.140 and AVFluIgG03. While increasing the resistance to FLD21.140 about 23-fold, the L133aS mutation transformed A/Vietnam/1203/2004 from completely resistant to highly sensitive to AVFluIgG03. Conversely, of the 27 single mutants in the A/Anhui/1/2005 envelope, three amino acid exchanges for those of A/Vietnam/1203/2004 (S133aL, T144K, and P145S) had the opposite effects on FLD21.140 and AVFluIgG03. By increasing the resistance to AVFluIgG03 about 5415-, 15-, and 25-fold, S133aL, T144K, and P145S mutants had an increased sensitivity to FLD21.140 by about 38-, 4-, and 211-fold respectively, compared with the original WT envelope. The remaining mutant envelopes demonstrated a similar trend of resistance to FLD21.140 and AVFluIgG03, although the actual levels varied from mutant to mutant. Finally, when the exchange mutations were tested in double and triple mutants, the distinct impact on FLD21.140 and AVFluIgG03 became even more pronounced. For instance, although the double mutants L133aS/K144T and L133aS/S145P of A/Vietnam/1203/2004 resulted in more than 137- and 1006-fold increases of resistance to FLD21.140, respectively, they significantly increased the sensitivity to AVFluIgG03. The impact of the triple mutant L133aS/K144T/S145P was the most significant (Table 3). Similarly, although the double mutant S133aL/P145S and the triple mutant S133aL/T144K/P145S of A/Anhui/1/2005 conferred complete resistance to AVFluIgG03, they increased the neutralization sensitivity to FLD21.140 more than 1000-fold. The antibodies' binding activities to the globular head proteins were also found to mirror their neutralizing activities (Figs. S2 and S3 and Table S5). Taken together, these results indicate that the residues at positions 133a, 144, and 145 play a critical role in determining the specificity of FLD21.140 and AVFluIgG03, whereas those at the remaining positions of the epitope were recognized by both antibodies with similar activity.

**Table 3:** **Summary of neutralization IC_50_ (μg/ml) of FLD21.140 and AVFluIgG03 against single, double, and triple mutants of A/Vietnam/1203/2004 and A/Anhui/1/2005 H5N1 pseudoviruses**

A/Vietnam/1203/2004					A/Anhui/1/2005				
Mutant	FLD21.140		AVFluIgG03		Mutant	FLD21.140		AVFluIgG03	
	IC_50_	Fold*^a^*	IC_50_	Fold*^a^*		IC_50_	Fold*^a^*	IC_50_	Fold*^a^*
WT	0.001	1.0	>50.000	/	WT	2.109	1.0	0.002	1.0
E131A	0.008	8.0	>50.000	/	E131A	>50.000	*^b^*	0.016	8.0
E131D	0.002	2.0	>50.000	/	E131D	16.718	7.9	0.624	312.0
S133A	0.003	3.0	>50.000	/	S133A	6.950	3.3	0.002	1.0
L133aS	0.023	23.0	0.270	*^c^*	S133aL	0.055	1/38.3	10.830	5415.0
L133aF	>50.000	*^b^*	>50.000	/	S133aF	NA	NA	NA	NA
G134A	NA*^d^*	NA	NA	NA	G134A	NA	NA	NA	NA
V135A	0.009	9.0	>50.000	/	V135A	40.120	19.0	0.002	1.0
V135W	0.027	27.0	>50.000	/	V135W	NA	NA	NA	NA
S136A	0.012	12.0	>50.000	/	S136A	4.851	2.3	0.015	7.5
S137A	0.007	7.0	>50.000	/	S137A	40.000	18.9	0.023	11.5
G143R	0.007	7.0	NA	NA	G143A	5.826	2.8	NA	NA
K144A	0.018	18.0	>50.000	/	T144A	4.524	2.1	0.002	1.0
K144T	0.020	20.0	>50.000	/	T144K	0.536	1/3.9	0.030	15.0
S145A	0.002	2.0	>50.000	/	P145A	0.130	1/16.2	0.002	1.0
S145P	0.014	14.0	>50.000	/	P145S	0.010	1/210.9	0.049	24.5
W153A	0.001	1.0	>50.000	/	W153A	>50.000	*^b^*	0.015	7.5
I155A	0.006	6.0	>50.000	/	I155A	>50.000	*^b^*	0.016	8.0
I155T	0.023	23.0	>50.000	/	I155T	22.029	10.4	0.019	9.5
K156A	0.005	5.0	>50.000	/	K156A	28.950	13.7	0.404	202.0
K157A	0.001	1.0	>50.000	/	K157A	4.163	2.0	0.010	5.0
N158A	0.002	2.0	>50.000	/	N158A	4.389	2.3	0.022	11.0
S159A	0.001	1.0	>50.000	/	N159A	24.400	11.6	0.178	89.0
S159N	0.017	17.0	>50.000	/	N159S	14.700	7.0	0.086	43.0
E190A	0.004	4.0	NA	NA	E190A	1.118	0.5	0.002	1.0
K193A	0.001	1.0	>50.000	/	K193A	9.995	4.7	1.170	585.0
L194A	0.001	1.0	>50.000	/	L194A	6.111	2.9	0.001	0.5
L194W	0.084	84.0	>50.000	/	L194W	NA	NA	NA	NA
K222A	NA	NA	NA	NA	K222A	NA	NA	0.005	2.5
G225A	NA	NA	NA	NA	G225A	NA	NA	0.013	6.5
Q226A	NA	NA	NA	NA	Q226A	NA	NA	0.007	3.5
L133aS/K144T	0.137	137.0	0.145	*^c^*	S133aL/T144K	0.002	1/1050.0	5.184	2592.0
L133aS/S145P	1.006	1006.0	0.023	*^c^*	S133aL/P145S	0.002	1/1050.0	>50.000	*^b^*
K144T/S145P	0.017	17.0	>50.000	/	T144K/P145S	0.027	1/78.1	0.021	10.5
L133aS/K144T/S145P	2.697	2697.0	0.002	*^c^*	S133aL/T144K/P145S	0.001	1/2100.0	>50.000	*^b^*

*^a^* Fold-change in the IC_50_ value of the mutant over the wildtype is shown.

*^b^* Data show sensitive to completely resistant.

*^c^* Data show completely resistant to sensitive.

*^d^* NA means not applicable.

### Naturally occurring mutations confirm the roles of the critical residues

We proceeded to study the impact of naturally occurring mutations at positions 133a, 144, and 145 on virus recognitions by the two antibodies. Based on the 3758 H5N1 HA sequences currently available in the database, including the panel of 18 diverse H5N1 strains initially used for characterization of the two antibodies (Fig. 3 and Table S4), we identified the degree of sequence variation at positions 133a, 144, and 145. Although dominated by two major residues at positions 133a (45% Leu and 43% Ser) and 145 (74% Ser and 26% Pro), significant levels of polymorphism were found at the position 144 (33% Arg, 25% Asn, 15% Lys, 8% Ser, and 7% Thr) (Table 4 and Table S4). Pseudoviruses bearing each polymorphic residue were then generated in the background of A/Vietnam/1203/2004 and A/Anhui/1/2005, confirmed by sequencing, and then subjected to sensitivity analysis to FLD21.140 and AVFluIgG03 neutralization. Of the 12 A/Vietnam/1203/2004 envelope clones tested, six (Ser-133a, Asn/Thr/Met/Val-144, and Pro-145) conferred more than 10-fold resistance to FLD21.140, compared with the original WT envelope (Table 4). In the case of the 12 A/Anhui/1/2005 envelope clones tested, three (Leu-133a, Lys-144, and Ser-145) became more than 10-fold resistant to AVFluIgG03. A closer examination of these resistance-conferring mutations confirmed that polymorphic residues at positions 133a (Ser and Leu) and 145 (Ser and Pro) had clearly opposite effects on the two antibodies, whereas those at position 144 were more complex. For instance, in the context of A/Vietnam/1203/2004, K144N/K144T/K144M/K144V mutations increased the level of resistance to FLD21.140 but had minimal effects on AVFluIgG03 (Table 4, left panel). In the context of A/Anhui/1/2005, however, only T144K had a significant impact on AVFluIgG03, and the remaining mutations maintained their sensitivity to both antibodies (Table 4, right panel). These results further reinforced the critical roles of naturally occurring residues at positions 133a, 144, and 145 in determining the specificity of the two antibodies. Contributions from the residues at positions 133a and 145, however, appear to be more important than those at position 144 given the variable degree of polymorphism and impact of particular mutations on the neutralization sensitivity to the two antibodies.

**Table 4:** **Summary of neutralization IC_50_ (μg/ml) of FLD21.140 and AVFluIgG03 against wildtype A/Vietnam/1203/2004 and A/Anhui/1/2005 H5N1 pseudoviruses and mutants constructed based on mutations existing in nature in the context of the two HAs at positions 133a, 144, and 145**

A/Vietnam/1203/2004						A/Anhui/1/2005					
Position	Amino acids	FLD21.140		AVFluIgG03		Position	Amino acids	FLD21.140		AVFluIgG03	
		IC_50_	Fold*^a^*	IC_50_	Fold*^a^*			IC_50_	Fold*^a^*	IC_50_	Fold*^a^*
133a	L (45.07%)	0.001	–*^b^*	>50.000	–*^b^*	133a	L (45.07%)	0.055	1/38.3	10.830	5415.0
	S (42.67%)	0.023	23.0	0.270	*^c^*		S (42.67%)	2.109	–*^b^*	0.002	–*^b^*

144	R (33.47%)	0.003	3.0	>50.000	/	144	R (33.47%)	0.667	1/3.2	0.001	0.5
	N (25.37%)	0.024	24.0	>50.000	/		N (25.37%)	7.450	3.5	0.002	1.0
	K (15.12%)	0.001	–*^b^*	>50.000	–*^b^*		K (15.12%)	0.536	1/3.9	0.030	15.0
	S (8.05%)	0.005	5.0	32.210	*^c^*		S (8.05%)	3.030	1.4	0.002	1.0
	T (7.32%)	0.020	20.0	>50.000	/		T (7.32%)	2.109	–*^b^*	0.002	–*^b^*
	G (3.74%)	0.002	2.0	49.670	*^c^*		G (3.74%)	0.563	1/3.7	0.002	1.0
	Q (2.63%)	0.002	2.0	35.920	*^c^*		Q (2.63%)	1.039	0.5	0.006	3.0
	M (1.76%)	0.010	10.0	>50.000	/		M (1.76%)	0.578	1/3.6	0.002	1.0
	V (1.20%)	0.019	19.0	>50.000	/		V (1.20%)	0.422	1/5.0	0.003	1.5
	E (0.70%)	0.001	1.0	>50.000	/		E (0.70%)	11.979	5.7	0.004	2.0

145	P (25.76%)	0.014	14.0	>50.000	/	145	P (25.76%)	2.109	–*^b^*	0.002	–*^b^*
	S (73.62%)	0.001	–*^b^*	>50.000	–*^b^*		S (73.62%)	0.010	1/210.9	0.049	24.5

*^a^* Fold-change in the IC_50_ value of the mutant over the wildtype is shown.

*^b^* Dashes indicate the amino acid at the site is naturally present in wildtype HAs.

*^c^* Data show from completely resistant to sensitive.

### Mutant residues conferring resistance against mouse antibodies and airborne transmission between ferrets are well tolerated by FLD21.140 and AVFluIgG03

Next, we asked whether the resistant mutants generated by the mouse monoclonal antibodies and immune serum could generate any cross-resistance against FLD21.140 and AVFluIgG03. To this end, we generated an additional 22 escape mutants based on exactly the same as published on the backbone of the WT H5N1 HA-expressing clones such as A/Vietnam/1203/2004 WT, A/HongKong/487/1997 WT, A/Indonesia/5/2005 WT, A/Turkey/65596/2006 WT, and A/Chicken/Korea/ES/2003 WT (13, 51–62). Pseudoviruses bearing these mutations were confirmed by sequencing and then subjected to sensitivity analysis to FLD21.140 and AVFluIgG03 neutralization. As shown in Table 5, FLD21.140 and AVFluIgG03 demonstrated strong neutralizing activities against these mutants and therefore a high degree of tolerance toward residue variations within and surrounding the RBS. More importantly, both antibodies also maintained equivalent or even stronger neutralizing potency against mutant H5N1 strains that acquired ability in respiratory droplets transmission among ferrets (Table 5) (8, 9, 48). These results suggest that FLD21.140 and AVFluIgG03 are capable of neutralizing almost all mutant H5N1 strains either induced in the mouse model or artificially generated *in vitro*. Such tolerance for mutations within and around RBS highlights their promising potential in inhibiting a broad array of H5N1 variants that either naturally occurred or were intentionally generated by humans.

**Table 5:** **Neutralization activity of FLD21.140 and AVFluIgG03 against the escape mutants and respiratory droplet transmission (RDT) mutants**

Clade	Escape mutant	FLD21.140		AVFluIgG03	
		IC_50_*^a^*	Fold*^b^*	IC_50_*^a^*	Fold*^b^*
**Escape mutants selected by mouse antibodies or immune serum**					
0	A/HongKong/487/1997 WT	0.281	1.0	>50.000	/
	A/HongKong/487/1997 D131E	0.009	0.1	0.055	*^c^*
1	A/Vietnam/1203/2004 WT	0.001	1.0	>50.000	/
	A/Vietnam/1203/2004 G143E	0.004	4.0	>50.000	/
	A/Vietnam/1203/2004 S145F	0.026	26.0	>50.000	/
	A/Vietnam/1203/2004 S145T	0.005	5.0	>50.000	/
	A/Vietnam/1203/2004 K156E	0.005	5.0	>50.000	/
	A/Vietnam/1203/2004 K156N	0.006	6.0	>50.000	/
	A/Vietnam/1203/2004 K156Q	0.002	2.0	>50.000	/
	A/Vietnam/1203/2004 S159I	0.004	4.0	>50.000	/
	A/Vietnam/1203/2004 K193E	0.038	38.0	>50.000	/
	A/Vietnam/1203/2004 K193T	0.002	2.0	>50.000	/
	A/Vietnam/1203/2004 K193N	0.004	4.0	>50.000	/
2.1.3.2	A/Indonesia/5/2005 WT	13.600	1.0	1.132	1.0
	A/Indonesia/5/2005 G143E	8.418	0.6	1.185	1.0
	A/Indonesia/5/2005 S159N	0.596	0.1	0.050	0.1
	A/Indonesia/5/2005 S159I	>50.000	*^d^*	>50.000	*^d^*
	A/Indonesia/5/2005 S159D	7.641	0.6	4.130	3.6
	A/Indonesia/5/2005 R193M	18.634	1.4	10.417	9.2
	A/Indonesia/5/2005 R193W	23.717	1.7	1.388	1.0
	A/Indonesia/5/2005 R193K	1.483	0.1	0.005	0.1
	A/Indonesia/5/2005 K222Q	1.904	0.1	0.822	0.7
2.2.1	A/Turkey/65596/2006 WT	0.001	1.0	1.861	1.0
	A/Turkey/65596/2006 R144M	0.044	44.0	1.756	0.9
	A/Turkey/65596/2006 R193K	0.005	5.0	0.012	0.1
2.5	A/Chicken/Korea/ES/2003 WT	0.034	1.0	16.667	1.0
	A/Chicken/Korea/ES/2003 G143R	0.007	0.2	6.904	0.4

**Respiratory droplet transmission (RDT) mutants**					
1	A/Vietnam/1203/2004 WT	0.001	1.0	>50.000	/
	N158D + N224K + Q226L	0.001	1.0	23.710	*^c^*
	T160A + Q226L + G228S	<0.001	<0.2	1.487	*^c^*
2.1.3.2	A/Indonesia/5/2005 WT	13.600	1.0	1.132	1.0
	N158D/N224K/Q226L	14.124	1.0	0.069	<0.1
	T160A/Q226L/G228S	4.378	0.3	0.042	<0.1
2.3.4	A/Anhui/1/2005 WT	2.109	1.0	0.002	1.0
	N158D/N224K/Q226L	5.976	2.8	0.001	0.5
	T160A/Q226L/G228S	0.420	0.2	<0.001	<0.001

*^a^* IC_50_(μg/ml) is the half-maximal inhibition concentration.

*^b^* Fold-change in the IC_50_ of the mutant over the wildtype is shown.

*^c^* Data show completely resistant to sensitive.

*^d^* Data show sensitive to completely resistant.

### Structural features of the critical residues

**Figure 4.**
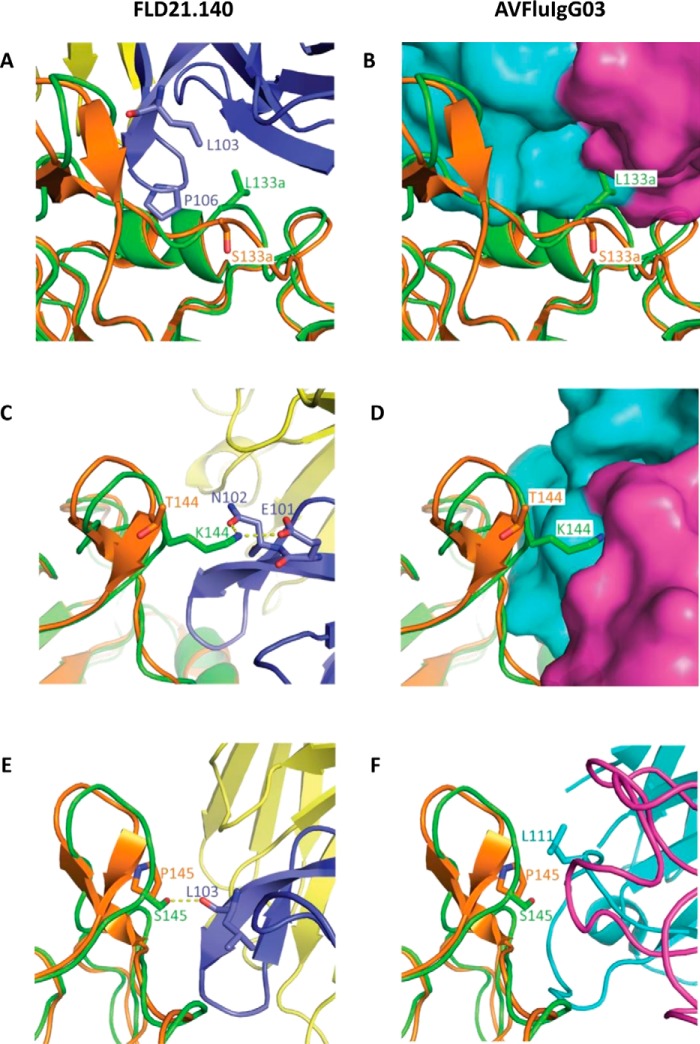
**Structural features of the critical residues 133a, 144, and 145 at the binding interface.** *A, C,* and *E,* spatial location of residues 133a, 144, and 145 on the globular head of A/Thailand/1(KAN-1)/2004 (*green*) and A/Anhui/1/2005 (*orange*) and their contacting residues on FLD21.140 HCDR3. *B, D,* and *F,* spatial location of residues 133a, 144, and 145 on the globular head of A/Anhui/1/2005 and A/Thailand/1(KAN-1)/2004, and their contacting residues on AVFluIgG03.

To investigate how the critical residues 133a, 144, and 145 determined the differences of antibody recognition, we conducted a more in-depth structural analysis of their interactions with FLD21.140 and AVFluIgG03 (Fig. 4). In the context of FLD21.140 recognition of A/Thailand/1(KAN-1)/2004, the residue Leu-133a of the HA globular head mainly formed hydrophobic interactions with Leu-103 and Pro-106 of FLD21.140 (Fig. 4*A*). However, replacing the leucine with serine, such as in the A/Anhui/1/2005, introduced a hydrophilic residue, which would disrupt the hydrophobic interactions around this position (Fig. 4*A*). By contrast, for AVFluIgG03 recognition of A/Anhui/1/2005, the S133aL mutation introduced a larger side chain, which might lead to steric clashes between HA and AVFluIgG03 (Fig. 4*B*). The Leu-133a and Ser-133a residues were therefore favored by FLD21.140 and AVFluIgG03 in their respective recognition. Furthermore, the residue Lys-144 on the HA globular head of A/Thailand/1(KAN-1)/2004 formed a salt bridge and a hydrogen bond with Glu-101 and Asn-102 of FLD21.140 HCDR3, respectively (Fig. 4*C*). The K144T mutation introduced a short and uncharged side chain, thereby disfavoring the antibody binding at this position (Fig. 4*C*). Conversely, although AVFluIgG03 recognized Thr-144 on the globular head of A/Anhui/1/2005, the T144K mutation would introduce a long side chain that was disfavored by AVFluIgG03 (Fig. 4*D*). Lys-144 and Thr-144 were therefore distinctly favored by FLD21.140 and AVFluIgG03, respectively. As for the residue at position 145, Ser-145 on the HA globular head of A/Thailand/1(KAN-1)/2004 was required for the formation of a hydrogen bond with the main chain oxygen atom of Leu-103 in FLD21.140 (Fig. 4*E*). Replacement of Ser-145 with Pro-145 from A/Anhui/1/2005 would disrupt the hydrogen bond and disfavor recognition by FLD21.140. By contrast, Pro-145 on the HA globular head of A/Anhui/1/2005 was required for the hydrophobic interaction with Leu-111 of AVFluIgG03 (Fig. 4*F*). Thus, the P145S mutation, while in favor of FLD21.140 recognition, would also disfavor AVFluIgG03 recognition. Taken together, these results highlighted the unique structural basis for the roles of the residues at positions 133a, 144, and 145 in the differential recognition by the two antibodies. Because only the residue 133a was located within the RBS, the distinct recognition between FLD21.140 and AVFluIgG03 was determined by residues both within and outside of the RBS.

## Discussion

**Figure 5.**
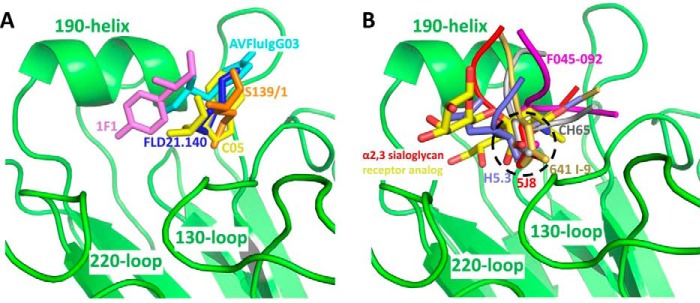
**FLD21.140 and AVFluIgG03 utilize a hydrophobic loop-insertion binding mode to block α2,3-sialoglycan receptor binding.** A structural comparison of antibodies directed against the receptor-binding site and an α2,3-sialoglycan receptor analog revealed that FLD21.140 and AVFluIgG03 utilize a hydrophobic loop-insertion binding mode to block α2,3-sialoglycan receptor binding (*A*) instead of mimicking receptor binding (*B*). (FLD21.140: *blue*; AVFluIgG03: *cyan*, PDB code 5DUP; 1F1: *violet*, PDB code 4GXU; C05: *yellow*, PDB code 4FP8; S139/1: *orange*, PDB code 4GMS; H5.3: *slate*, PDB code 4XNM; 5J8: *red*, PDB code 4M5Z; 641 I-9: *yellow-orange*, PDB code 4YK4; CH65: *gray*, PDB code 5UGY; F045-092: *magenta*, PDB code 4O58; α2,3-sialoglycan receptor analog: carbon atoms in *red* and oxygen atoms in *yellow* PDB code 4BGY.)

RBS-specific antibodies play critical roles in protecting the host from influenza virus infection. They are by far the most predominant among the isolated antibodies and perhaps the best characterized despite their structural and functional diversity (34, 42–49). Identifying their unique as well as common features will greatly improve our understanding of their protective role *in vivo* and guide the future design of more effective vaccines against influenza virus infection. Over the last few years, while studying a panel of human monoclonal antibodies isolated from H5N1-infected and convalescent patients, we identified the two antibodies FLD21.140 and AVFluIgG03 that are mutually complementary in their binding and neutralizing activities against a diverse panel of H5N1 strains. In this study, we used structural and mutational studies, which uncovered that both FLD21.140 and AVFluIgG03 recognize the RBS through insertion of their HCDR3 region in a manner that directly competes with the sialic acid receptor. Such a mode of recognition is rather common, and only one exception has so far been reported, where the HCDR2 rather than the HCDR3 region mediated the recognition (49). Furthermore, two major mechanisms of recognition have been postulated, one relying on hydrophobic interaction and the other on receptor mimicry (Fig. 5). The FLD21.140 and AVFluIgG03 antibodies, together with 1F1, C05, and S139/1, fall into the former category (Fig. 5*A* and Table S6), in which the recognition is mediated through hydrophobic interactions in the conserved hydrophobic cavity. Other antibodies, such as H5.3, 5J8, 641 I-9, CH65, and F045-092, rely on receptor mimicry in which an aspartic acid side chain on the tip of HCDR3 forms contacts similar to those made by the carboxylic acid of the receptor (Fig. 5*B* and Table S6). However, it should be noted that the potency and breadth of these antibodies varies despite their shared specificity and mechanism of recognition against RBS. This is largely dependent on the degree of residue variation in their recognition sites. For instance, antibodies with broader and cross-clade activity such as C05, S139/1, and F045-092 tend to recognize more conserved residues (45, 47, 49), whereas subtype- and strain-specific antibodies such as FLD21.140, AVFluIgG03, H5.3, 5J8, 1F1, 641 I-9, and CH65 recognize the more polymorphic ones both within and beyond the RBS (34, 39, 42–44, 46, 48). Indeed, the unique complementarity between FLD21.140 and AVFluIgG03 in binding and neutralization can be explained by their specific and preferential recognition of residues at positions 133a, 144, and 145 located within and beyond the RBS. Although FLD21.140 clearly prefers the residue combination Leu-133a/Lys-144/Ser-145, AVFluIgG03 favors Ser-133a/Thr-144/Pro-145 instead.

**Figure 6.**
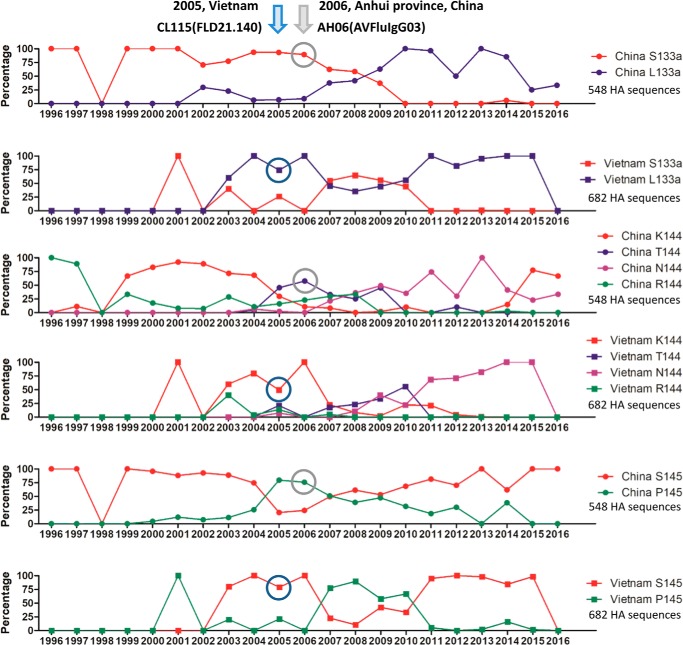
**Fluctuation of amino acid residues at positions 133a, 144, and 145 over time.** The temporal changes in residue composition for Ser/Leu-133a, Lys/Thr/Asn/Arg-144, and Ser/Pro-145 are indicated by different colors based on 548 HA sequences from China and 682 sequences from Vietnam collected between 1996 and 2016. The years when FLD21.140 and AVFluIgG03 were isolated are indicated on the *top*, and their representations among analyzed sequences are *circled*.

As in the development of antibodies against any targets, the specific recognition of FLD21.140 and AVFluIgG03 is likely to result from distinct antibody responses against different infecting virus strains in patients from whom the two antibodies were initially isolated. Indeed, FLD21.140 was isolated in 2005 from the Vietnamese patient CL115 infected with the clade 1 strain A/Vietnam/CL115/2005, whereas AVFluIgG03 was isolated in 2006 from the Chinese patient AH06 infected with the clade 2.3.4 strain A/Anhui/1/2005 (38, 39). An analysis of temporal changes in residue sequences at positions 133a, 144, and 145 revealed a perfect match with the specificities of FLD21.140 and AVFluIgG03 (Fig. 6). When FLD21.140 and AVFluIgG03 were isolated, the dominant residues among the 682 HA sequences from Vietnam were Leu-133a/Lys-144/Ser-145, compared with Ser-133a/Thr-144/Pro-145 among the 548 HA sequences from China (Fig. 6). However, substantial fluctuations in residue sequence were found at all three positions over the last 20 some years since the first discovery of H5N1 in a sick goose in Guangdong during the summer of 1996 (63). Residues at positions 133a and 145 were predominantly represented by two residues, whereas position 144 was more diverse (Fig. 6). It is expected that antibody specificity would react accordingly to changes in viral sequences and fluctuate along the way. Obviously, such a rapid turnover of viral sequences in the RBS enables viral escape from immune recognition and implies the necessity to appropriately change the influenza vaccines to match the predicted circulating strains. In this regard, vaccine developed based on clade 1 A/Vietnam/CL115/2005 from Vietnam would be unsuitable and insufficient against a clade 2.3.4 strain A/Anhui/1/2005 from China and vice versa. Furthermore, analysis of the 2016–2017 circulating H5N1 HA sequences from National Center for Biotechnology Information (NCBI) reveals that an interesting convergence is happening among the H5N1 strains circulating in China and Vietnam, whereby novel combinations of Met-133a/Lys-144/Ser-145 and Leu-133a/Asn-144/Ser-145 appear to be respectively dominant in China and Vietnam over the last few years. Individuals infected with a novel H5N1 strain bearing such a combination would be expected to develop a novel class of RBS-specific antibodies with different specificity from FLD21.140 and AVFluIgG03. However, it is encouraging to realize that many of the mutants induced by mouse antibodies and serum had little impact on neutralizing activity of FLD21.140 and AVFluIgG03. More importantly, FLD21.140 and AVFluIgG03 demonstrated equivalent or even greater neutralizing activity against mutants that acquired transmission ability through respiratory droplets among ferrets. All these results pointed to the critical role of FLD21.140 and AVFluIgG03 and other RBS-specific antibodies in inhibiting influenza virus infection and promising potential in prophylactic and therapeutic interventions against H5N1 infection in humans.

## Experimental procedures

### H5N1 antibodies

The antibody FLD21.140 was isolated from the Vietnamese donor CL115 who had recovered from an infection with the HPAI H5N1 virus in 2005 (39). The antibody AVFluIgG03 was isolated by screening a Fab antibody phage library from the Chinese patient AH06 who survived an H5N1 virus infection in 2006 (38). The VH and VL genes of FLD21.140 were synthesized based on the reported sequences and cloned into the backbone of the IgG1 antibody expression vectors containing the constant regions of human IgG1 (provided by M. C. Nussenzweig, Rockefeller University). The AVFluIgG03 expression plasmid was kindly provided by Professor Mifang Liang from the China CDC, Beijing. Production of full-length human IgG1 was conducted by transient transfection of HEK293T cells (human embryonic kidney cells 293T, obtained from the ATCC). The expressed antibodies were purified by affinity chromatography using protein A-agarose (Pierce, ThermoFisher Scientific) and titrated using a BCA Protein Assay Kit (ThermoFisher Scientific).

### Production and purification of the H5N1 HA WT and mutated globular head proteins

The H5N1 (A/Thailand/1(KAN-1)/2004, A/Vietnam/1203/2004, A/Turkey/65596/2006, A/Anhui/1/2005, A/Beijing/01/2003) HA globular head proteins (residues Asp-55–Glu-271) were expressed in Sf9 insect cells (*Spodoptera frugiperda* 9, obtained from the ATCC) using the Bac-to-Bac baculovirus expression system (Invitrogen). The genes encoding the globular head were cloned into the pFastBac-Dual vector (Invitrogen) with an N-terminal gp67 signal peptide for secretion and a C-terminal His_6_ tag for purification. Specifically, the plasmids were first introduced into DH10Bac competent cells, and the bacmids were used to transfect Sf9 insect cells using Cellfectin II reagent (Invitrogen). The recombinant baculovirus virions were harvested after 7 days. After one round of amplification, the high-titer virions were used to infect Sf9 insect cells at a density of 2 × 10^6^ per ml. After 60 h of infection, the secreted globular head was harvested, concentrated, and buffer-exchanged to HBS buffer (10 mm HEPES, pH 7.2, 150 mm NaCl). The globular head was captured on nickel-nitrilotriacetic acid–agarose beads (GE Healthcare), eluted with 500 mm imidazole in HBS buffer, and then purified through gel-filtration chromatography using a Superdex 200 column (GE Healthcare). The H5N1 HA mutated globular heads of A/Vietnam/1203/2004 bearing L133aS/K144T, L133aS/S145P, K144T/S145P, and L133aS/K144T/S145P mutations, as well as of A/Anhui/1/2005 bearing S133aL/T144K, S133aL/P145S, T144K/P145S, and S133aL/T144K/P145S mutations were produced and purified in the same way as the WT globular head.

### ELISA

Reactivity of human antibodies (FLD21.140 and AVFluIgG03) against the five tested HA globular head (Asp-55–Glu-271) antigens was determined by ELISA. Briefly, 100 μl of purified HA globular heads (1 μg/ml) were used to coat 96-well ELISA plates (Biofil, China) at 4 °C overnight. The plates were blocked with 10% bovine serum albumin or fetal bovine serum for 2h at 37 °C and then washed with PBS, 0.05% Tween 20 (PBST) three times. The tested human antibodies (diluted to 10 μg/ml) were added into the wells and incubated at 37 °C for 1 h, followed by washing with PBST. The antibodies were serially diluted 3-fold. Bound antibodies were detected with HRP-conjugated goat anti-human IgG (Promega) at 37 °C for 1 h, followed by washing. The reaction was visualized by addition of 100 μl of 3,3′,5,5′-tetramethylbenzidine substrate (CWBio, China), and stopped by the addition of 50 μl 0.5 m H_2_SO_4_. The absorbance at 450 nm was measured using a ELISA plate reader (Bio-Rad).

### Affinity measurement via surface plasmon resonance (SPR)

The binding kinetics and affinity of FLD21.140 and AVFluIgG03 to purified HA globular heads (Asp-55–Glu-271) were analyzed by SPR (Biacore T200, GE Healthcare). The purified soluble globular head was covalently immobilized to a CM5 sensor chip via amine groups using the amine coupling kit (Biacore) in 10 mm sodium acetate buffer, pH 5.0. Both antibodies were cleaved to generate antigen-binding fragments (Fabs) by incubating with protease Lys-C (Sigma) at an IgG/Lys-C ratio of 4000:1 (w/w) in 10 mm EDTA, 100 mm Tris/HCl, pH 8.5, at 37 °C for 12 h. SPR experiments were run at a flow rate of 30 μl/min in HBST buffer (10 mm HEPES, pH 7.2, 150 mm NaCl, 0.005% Tween 20). The surface was regenerated with 10 mm NaOH. The sensorgrams were fit with a 1:1 binding model using BIA Evaluation software (GE Healthcare).

### Generation of H5N1 WT and mutated pseudoviruses

Table 1 lists 18 H5N1 pseudoviruses used in this study. The H5 panel covers all 10 clades and 5 subclades of clade 2 of H5N1 virus. The HA plasmids, NA plasmids, and backbone plasmids used for generating H5N1 pseudoviruses were kindly provided by Professor Paul Zhou from the Institute Pasteur of Shanghai, Chinese Academy of Sciences. Pseudoviruses bearing the WT HA glycoprotein from all 18 strains of H5N1 were constructed as reported previously (41, 64). Pseudoviruses with single or multiple point mutations on the HA gene were generated by site-directed mutagenesis according to the instruction manual of the QuikChange II site-directed mutagenesis kit (200523; Stratagene). The numbering system used in this paper was based on H3 viruses. To improve the production of pseudoviruses, HAs codon-optimized for eukaryotic cell expression were used throughout the experiments.

### Pseudovirus-based neutralization assay

The pseudovirus-based neutralization assay was conducted as described previously (41, 64). Briefly, serial 3-fold dilutions of each antibody were incubated with pseudovirus particles in a final volume of 100 μl at 37 °C for 1 h. Madin-Darby canine kidney cells (5 × 10^3^–10^4^ cells per well; obtained from ATCC) were then added to each antibody/virus mixture. The relative luciferase activity was measured after 48 h using the BrightGlo luciferase assay (Promega), according to the manufacturer's instructions. Titration curves were generated using sigmoid dose response with nonlinear fit in GraphPad Prism software version 5, and inhibitory concentration (IC_50_) values were determined in the region in which a 50% reduction of relative luciferase activity was identified.

### Flow cytometry analysis of WT and mutated HA binding by the FLD21.140 and AVFluIgG03 antibodies

The expression plasmids encoding the genes of WT and mutant HAs were used to transiently transfect HEK293T cells. Approximately 40 h later, the transfected cells were harvested, trypsinized, fixed with 4% paraformaldehyde, stained with FLD21.140 and AVFluIgG03 antibodies at a concentration of 10 μg/ml, and detected using an anti-human IgG-Alexa Fluor 488 secondary antibody (Santa Cruz Biotechnology) using a FACSCalibur flow cytometer (BD Biosciences).

### Production of the FLD21.140 Fab and the globular head complex for crystallization

The purified FLD21.140 IgG was digested using papain from papaya latex (P3125; Sigma) at 37 °C for 12 h, and the digested Fab and Fc fragments were separated by loading the mixture onto a protein A column. The Fab fragment was collected in the flow-through, and the Fc fragment was captured on the protein A beads. The flow-through was further purified by gel filtration (Superdex 200 column, GE Healthcare). The purified globular heads (A/Thailand/1(KAN-1)/2004) and FLD21.140 Fab were mixed at a molar ratio of 1:1, incubated at 4 °C for 1 h, and then purified by gel-filtration chromatography on a Superdex 200 column (GE Healthcare). For crystallization, the globular head–FLD21.140 Fab complex was concentrated to 20 mg/ml in HBS buffer. Crystals were successfully grown at 18 °C using the vapor diffusion method in sitting drops, which consisted of equal volumes of protein and reservoir solution containing 2.0 m (NH_4_)_2_SO_4_ and 0.1 m BisTris, pH 5.5. All crystals were cryoprotected by soaking in reservoir solution with 20% v/v glycerol for several seconds and then frozen in liquid nitrogen before data collection. Diffraction data were collected on the BL17U beamline at the Shanghai Synchrotron Research Facility (SSRF) (65) and processed using the program HKL2000 (66). All data collection and processing statistics are listed in Table S2.

### Structure determination and refinement

The structure of the globular head–Fab complex was determined by the molecular replacement method using PHASER in CCP4 suite (67). The search models were the globular head of HA (A/Anhui/1/2005) (PDB code 5DUT) and structures of the variable and constant domains of heavy and light chains available in the Protein Data Bank with the highest sequence identities. Iterative refinements using PHENIX and model building with COOT were performed to complete the structural refinement (68, 69). Structure validation was performed using PROCHECK (70). All structure refinement statistics are listed in Table S2.

### Measurement of binding kinetics using bio-layer interferometry

The binding kinetics of FLD21.140 and AVFluIgG03 IgGs to the WT and mutant globular heads were measured via bio-layer interferometry using an Octet Red96 instrument (FortéBio, Inc.). The FLD21.140 and AVFluIgG03 IgGs diluted at 10–20 μg/ml in kinetics buffer (10 mm HEPES, pH 7.2, 150 mm NaCl, 0.01% Tween 20) were loaded onto protein A sensors and incubated with varying concentrations of WT and mutant globular heads in the same buffer. All the assays were carried out with agitation set to 1000 rpm at 25 °C. The experiments included six steps: 1) baseline (60 s); 2) IgG loading onto the protein A sensors for 120–200 s; the threshold of capture levels was set to 1 or 3 nm; 3) second baseline (120 s); 4) association of the globular head for the measurement of *k*_on_ (60–300 s); 5) dissociation of the globular head for the measurement of *k*_off_ (60–300 s); 6) regeneration and neutralization. The protein A sensors were regenerated for 5 s in regeneration buffer (10 mm glycine, pH 1.7) and then neutralized for 5 s in kinetics buffer. This was repeated three times. Five concentrations of heads were used, including the highest and lowest concentrations. Data processing and analysis were performed using Octet analysis software version 9.0 (FortéBio, Inc.). Experimental data were fitted using a 1:1 binding model for all assays. All sensorgrams used for fitting of *k*_on_ and *k*_off_ as well as the steady-state analysis curves are shown in Fig. S3.

## Author contributions

Y. Z., P. W., T. Z., X. W., and L. Z. conceptualization; Y. Z., P. W., S. G., and X. W. data curation; Y. Z., P. W., and S. G. software; Y. Z., P. W., S. G., and L. Z. formal analysis; Y. Z., P. W., X. W., and L. Z. validation; Y. Z., P. W., J. S., and G. W. investigation; Y. Z., P. W., X. W., and L. Z. visualization; Y. Z., P. W., J. S., S. G., G. W., and S. F. methodology; Y. Z., P. W., X. W., and L. Z. writing-original draft; Y. Z., P. W., X. W., and L. Z. writing-review and editing; P. Z. and M. L. resources; X. S. project administration; X. W. and L. Z. supervision; X. W. and L. Z. funding acquisition.

## Supplementary Material

Supporting Information

## Footnotes

5 Please note that the JBC is not responsible for the long-term archiving and maintenance of this site or any other third party hosted site.

## Footnotes

4 The abbreviations used are:

HPAI: highly-pathogenic avian influenza virus
RBS: receptor-binding site
HA: hemagglutinin
VS: vulnerable sites
SPR: surface plasmon resonance
HCDR: heavy chain complementarity determining region
PDB: Protein Data Bank
BisTris: 2-[bis(2-hydroxyethyl)amino]-2-(hydroxymethyl)propane-1,3-diol.
