# Supplementary material for: Complementary recognition of the receptor-binding site of highly pathogenic H5N1 influenza viruses by two human neutralizing antibodies
Source: J Biol Chem. 2018 Aug 28;293(42):16503–17. doi: 10.1074/jbc.RA118.004604 (PMC6200926; doi:10.1074/jbc.RA118.004604)
Supplement: Supporting Information [file supp_293_42_16503__index.html]

Complementary recognition of the receptor binding site of highly pathogenic H5N1 influenza viruses by two human neutralizing antibodies — Complementary human antibodies against H5N1 viruses — Complementary recognition of the receptor-binding site of highly pathogenic H5N1 influenza viruses by two human neutralizing antibodies — Complementary human antibodies against H5N1 viruses — Supporting Information 

# Complementary recognition of the receptor-binding site of highly pathogenic H5N1 influenza viruses by two human neutralizing antibodies

## Supporting Information

- Supporting Information (to be published online) - Supporting information containing supplementary tables and figures
