# Supplementary material for: Complementary recognition of the receptor-binding site of highly pathogenic H5N1 influenza viruses by two human neutralizing antibodies
Source: J Biol Chem. 2018 Aug 28;293(42):16503–17. doi: 10.1074/jbc.RA118.004604 (PMC6200926; doi:10.1074/jbc.RA118.004604)
Supplement: Supporting Information [file supp_RA118.004604_139020_1_supp_178123_pctccx.docx]

**Supporting Information**

Complementary recognition of the receptor binding site of highly pathogenic H5N1 influenza viruses by human neutralizing antibodies

Yanan Zuo(左亚男)^1¶^, Pengfei Wang(王鹏飞)^2¶^, Jianfeng Sun(孙建峰)^2^, Shichun Guo(郭世纯)^3^, Guiqin Wang(王桂芹)^4^, Teng Zuo(左腾)^1^, Shilong Fan(范仕龙)^2^, Paul Zhou(周保罗)^4^, Mifang Liang(梁米芳)^5^, Xuanling Shi(史宣玲)^1^, Xinquan Wang(王新泉) ^2*^, Linqi Zhang(张林琦)^1*^

^1^Comprehensive AIDS Research Center, Collaborative Innovation Center for Diagnosis and Treatment of Infectious Diseases, Department of Basic Medical Sciences, School of Medicine, Tsinghua University, Beijing 100084, China.

^2^The Ministry of Education Key Laboratory of Protein Science, Beijing Advanced Innovation Center for Structural Biology, Collaborative Innovation Center for Biotherapy, School of Life Sciences, Tsinghua University, Beijing 100084, China.

^3^Department of Computer Science, Tsinghua University, Beijing 100084, China.

^4^Unit of Anti-Viral Immunity and Genetic Therapy, Key Laboratory of Molecular Virology and Immunology, Institute Pasteur of Shanghai, Chinese Academy of Sciences, Shanghai 200031, China.

^5^State Key Laboratory for Infectious Disease Control and Prevention, National Institute for Viral Disease Control and Prevention, Chinese Center for Disease Control and Prevention, Beijing 102206, China.

**Table S1. Genetic analysis of FLD21.140 and AVFluIgG03.**

|  |  | FLD21.140 | AVFluIgG03 |
| --- | --- | --- | --- |
| Heavy chain | Germline V gene | 4-31*03 | 3-23*01 |
|  | Germline D gene | 3-3*02 | 4-23*01 |
|  | Germline J gene | 5*02 | 5*01 |
|  | Identity to germline gene(%) | 95.6 | 95.1 |
|  | CDR3 length | 17 | 20 |
| Light chain | Germline V gene | 1-44*01 | 1-40*01 |
|  | Germline J gene | 3*02 | 2*01 |
|  | Identity to germline gene(%) | 98.6 | 100 |
|  | CDR3 length | 12 | 9 |

**Table S2. X-ray data collection and refinement statistics.**

| FLD21.140 Fab/head | |
| --- | --- |
| **Data Collection** |  |
| Beamline | SSRF BL17U |
| Wavelength | 0.9796 Å |
| Space group | P4_2_22 |
| Cell dimensions |  |
| a, b, c (Å) | 168.09,168.09,147.19 |
| α, β, γ (°) | 90,90,90 |
| Resolution (Å) | 50.00-2.33 (2.37-2.33) |
| R_merge_^a^ | 0.091 (0.995) |
| R_pim_^b^ | 0.038 (0.425) |
| CC_1/2_^c^ | 0.999 (0.695) |
| I / σI | 22.3(2.6) |
| Completeness (%) | 99.5 (97.7) |
| Redundancy | 6.5 (6.0) |
| **Refinement** |  |
| Resolution (Å) | 36.50-2.33 |
| No. Reflections | 89830 |
| R_work_^d^ / R_free_ (%) | 19.7/23.8 |
| No. atoms |  |
| Protein  Glycan  water | 9876  56  414 |
| B-factors (Å^2^)  Protein  Glycan  water | 50.59  89.82  52.79 |
| r.m.s. deviations |  |
| Bond lengths (Å) | 0.010 |
| Bond angles (°) | 1.220 |
| Ramachandran plot (%)  Most favored  Additionally allowed  Generously allowed  Disallowed | 87.8  11.3  0.5  0.5 |

^a^*R_merge_ = ∑_hkl_ ∑_j_ |I_j_(hkl)-<I(hkl)>| / ∑_hkl_ ∑_j_ I_j_(hkl),* where *I* is the intensity of reflection.

^b^*R_pim_=∑_hkl_ [1/(N-1)]^1/2^∑_j_ |I_j_(hkl)-<I(hkl)>| / ∑_hkl_ ∑_j_ I_j_(hkl),* where *N* is the redundancy of the dataset.

^c^CC_1/2_ is the correlation coefficient of the half datasets.

^d^*R_work_ = ∑_hkl_ | |F_obs_| – |F_calc_| | / ∑_hkl_ |F_obs_|,* where *F_obs_* and *F_calc_* is the observed and the calculated structure factor, respectively. *R_free_* is the cross-validation *R* factor for the test set of reflections (5% of the total) omitted in model refinement.

**Table S3. Contacting residues at the binding interface (d ≤ 4 Å) between the globular head and the two antibodies, FLD21.140 and AVFluIgG03**.

|  | **FLD21.140** | | | | |  | **AVFluIgG03** | | | |
| --- | --- | --- | --- | --- | --- | --- | --- | --- | --- | --- |
|  | **Heavy chain** | | | **Light chain** | |  | **Heavy chain** | **Light chain** | | |
|  | **HCDR1** | **HCDR2** | **HCDR3** | **FR3** | **LCDR3** |  | **HCDR3** | **LCDR1** | **LCDR2** | **FR3** |
| **E131** |  | Y54;S56 |  |  |  | **E131** |  |  | G52;N53;S54 |  |
| **S133** | T33 | Y54 |  |  |  | **S133** |  | D34 |  |  |
| **L133a** | Y35 | Y54 | L103;P106 |  |  |  |  |  |  |  |
|  |  |  |  |  |  | **G134** | D104 |  |  |  |
| **V135** |  |  | L103;S105;P106 |  |  | **V135** | D104 |  |  |  |
| **S136** |  |  | L104 |  |  | **S136** | D104 |  |  |  |
| **S137** |  |  | L104 |  |  | **S137** | G107;Y109 |  |  |  |
| **R143** |  |  |  | Q54 |  |  |  |  |  |  |
| **K144** |  |  | E101;N102; |  |  | **T144** | L111 |  |  |  |
| **S145** |  |  | L103 |  |  | **P145** | Y109;L111 |  |  |  |
| **W153** |  |  | P106 |  |  | **W153** | D104 |  |  |  |
| **I155** |  |  | P106 |  |  | **I155** | Y103 |  |  |  |
| **K156** |  | S58;Y60 |  |  |  | **K156** |  |  |  | N55 |
|  |  |  |  |  |  | **K157** |  |  | S54 |  |
| **N158** |  | S56;G57;S58 |  |  |  | **N158** |  |  | S54;N55 | S65 |
| **S159** |  | S58 |  |  |  | **N159** |  |  | S54 | N55;R56 |
| **A189** |  |  |  |  | S97 |  |  |  |  |  |
| **E190** |  |  | Y107 |  |  |  |  |  |  |  |
| **K193** |  | Y60 | Y107 |  | S97 | **K193** | S102;Y103 |  |  |  |
| **L194** |  |  | P106;Y107 |  |  | **L194** | Y103 |  |  |  |
|  |  |  |  |  |  | **K222** | G106 |  |  |  |
|  |  |  |  |  |  | **G225** | G106;H108 |  |  |  |
|  |  |  |  |  |  | **Q226** | D104;G106;G107 |  |  |  |

**Table S4. Residue polymorphism within the epitopes of FLD21.140 and AVFluIgG03.** The common epitopes that are shared by both FLD21.140 and AVFluIgG03 are shown in dark gray.

| Pos | A % | B % | C % | D % | E % | F % | G % | H % | I % | K % | L % | M % | N % | P % | Q % | R % | S % | T % | V % | W % | X % | Y % |
| --- | --- | --- | --- | --- | --- | --- | --- | --- | --- | --- | --- | --- | --- | --- | --- | --- | --- | --- | --- | --- | --- | --- |
| 131 | 0.03 | 0 | 0 | 3.52 | 95.84 | 0 | 0.14 | 0 | 0 | 0 | 0 | 0 | 0.34 | 0 | 0 | 0 | 0 | 0 | 0 | 0 | 0 | 0.11 |
| 133 | 0 | 0 | 0 | 0 | 0 | 0.53 | 0 | 0 | 0 | 0 | 0 | 0 | 0 | 0.03 | 0 | 0 | 99.11 | 0.20 | 0 | 0 | 0 | 0 |
| 133a | 0.17 | 0 | 0 | 0 | 0 | 0 | 0.11 | 0 | 0 | 0.06 | 45.07 | 1.12 | 0 | 0 | 0 | 0 | 42.67 | 0 | 0 | 0 | 0 | 0 |
| 134 | 0 | 0 | 0 | 0 | 0 | 0 | 99.47 | 0 | 0 | 0 | 0 | 0 | 0 | 0.31 | 0 | 0.08 | 0 | 0 | 0 | 0.06 | 0 | 0 |
| 135 | 0.03 | 0 | 0 | 0 | 0 | 0 | 0.06 | 0 | 0 | 0 | 0 | 0.08 | 0 | 0 | 0 | 0 | 0 | 0 | 99.80 | 0 | 0 | 0 |
| 136 | 0 | 0 | 0 | 0 | 0 | 0 | 0.03 | 0 | 0 | 0 | 0 | 0 | 0 | 0 | 0 | 0.06 | 99.89 | 0 | 0 | 0 | 0 | 0 |
| 137 | 32.91 | 0 | 0 | 0 | 0 | 0 | 0 | 0 | 0 | 0 | 0.03 | 0 | 0 | 0 | 0 | 0 | 66.97 | 0.03 | 0 | 0 | 0.03 | 0 |
| 143 | 0 | 0 | 0 | 0.03 | 0.75 | 0 | 98.97 | 0 | 0 | 0 | 0 | 0 | 0 | 0 | 0 | 0.22 | 0 | 0 | 0 | 0 | 0 | 0 |
| 144 | 0.28 | 0 | 0 | 0.11 | 0.70 | 0 | 3.74 | 0 | 0.22 | 15.12 | 0 | 1.76 | 25.37 | 0 | 2.63 | 33.47 | 8.05 | 7.32 | 1.20 | 0 | 0 | 0 |
| 145 | 0.08 | 0 | 0 | 0 | 0 | 0.06 | 0 | 0.03 | 0 | 0 | 0.22 | 0 | 0 | 25.79 | 0 | 0 | 73.62 | 0.17 | 0 | 0 | 0 | 0 |
| 153 | 0 | 0 | 0 | 0 | 0 | 0 | 0.03 | 0 | 0 | 0 | 0 | 0 | 0 | 0 | 0 | 0 | 0 | 0 | 0 | 99.94 | 0 | 0 |
| 155 | 0 | 0 | 0 | 0 | 0 | 0 | 0 | 0 | 86.9 | 0 | 0.84 | 0 | 0 | 0 | 0 | 0 | 0 | 11.85 | 0.39 | 0 | 0 | 0 |
| 156 | 0 | 0 | 0 | 0 | 0.25 | 0 | 0.03 | 0 | 0.03 | 99.25 | 0 | 0 | 0.06 | 0 | 0.28 | 0 | 0 | 0.08 | 0 | 0 | 0 | 0 |
| 157 | 0 | 0 | 0 | 0 | 0 | 0 | 0 | 0 | 0 | 99.58 | 0 | 0 | 0 | 0 | 0 | 0.36 | 0 | 0 | 0 | 0 | 0.03 | 0 |
| 158 | 0 | 0.06 | 0 | 34.12 | 0.03 | 0 | 0.7 | 0 | 0 | 0.03 | 0 | 0 | 64.91 | 0 | 0 | 0 | 0.11 | 0 | 0 | 0 | 0.03 | 0 |
| 159 | 0.36 | 0 | 0 | 14.98 | 0 | 0 | 0.28 | 0.03 | 0 | 0 | 0 | 0 | 59.15 | 0 | 0 | 0.03 | 25.09 | 0.06 | 0 | 0 | 0 | 0 |
| 189 | 94.24 | 0 | 0 | 0 | 3.24 | 0 | 0 | 0 | 0 | 0.11 | 0 | 0 | 0 | 0 | 0 | 0 | 0 | 2.04 | 0.34 | 0 | 0 | 0 |
| 190 | 0.03 | 0 | 0 | 0 | 99.8 | 0 | 0 | 0.03 | 0 | 0.08 | 0 | 0 | 0 | 0 | 0 | 0 | 0 | 0 | 0 | 0 | 0.03 | 0 |
| 193 | 0 | 0 | 0 | 0.53 | 0.25 | 0 | 0.53 | 0 | 0 | 33.92 | 0.03 | 3.30 | 0.84 | 0 | 0.47 | 59.18 | 0.20 | 0.73 | 0 | 0 | 0 | 0 |
| 194 | 0 | 0 | 0 | 0 | 0 | 0 | 0 | 0 | 4.16 | 0 | 95.70 | 0 | 0 | 0 | 0 | 0 | 0.03 | 0 | 0.08 | 0 | 0 | 0 |
| 222 | 0 | 0 | 0 | 0 | 0.08 | 0 | 0 | 0 | 0 | 98.44 | 0 | 0 | 0 | 0 | 0.95 | 0.47 | 0 | 0.03 | 0 | 0 | 0 | 0 |
| 225 | 0 | 0 | 0 | 0 | 0.06 | 0 | 99.83 | 0 | 0 | 0 | 0 | 0 | 0 | 0 | 0 | 0.08 | 0 | 0 | 0 | 0 | 0 | 0 |
| 226 | 0 | 0 | 0 | 0 | 0 | 0 | 0 | 0 | 0 | 0.03 | 0.20 | 0 | 0 | 0.03 | 99.66 | 0.06 | 0 | 0 | 0 | 0 | 0 | 0 |

**Table S5. Binding affinity of FLD21.140 and AVFluIgG03 to the wild-type and double and triple mutants at positions 133a, 144 and 145 of A/Vietnam/1203/2004 (VN for short) and A/Anhui/1/2005 (AH for short) HA globular heads (D55-E271).**

| **HA globular head** | **FLD21.140** | |  | **AVFluIgG03** | |
| --- | --- | --- | --- | --- | --- |
| **(D55-E271)** | **KD(M)** | **Mut/WT KD Fold** |  | **KD(M)** | **Mut/WT KD Fold** |
| **VN WT** | 8.210E-11 | 1.0 |  | No binding | / |
| **VN L133aS/K144T** | 2.100E-6 | 25575.0 |  | 2.500E-6 | * |
| **VN L133aS/S145P** | 5.000E-6 | 60894.0 |  | 3.213E-8 | * |
| **VN K144T/S145P** | 7.921E-8 | 965.0 |  | No binding | / |
| **VN L133aS/K144T/S145P** | 1.500E-5 | 182682.0 |  | 4.227E-8 | * |
| **AH WT** | 8.900E-6 | 1.0 |  | 3.729E-10 | 1.0 |
| **AH S133aL/T144K** | 3.393E-9 | 1/2623.0 |  | No binding | ^#^ |
| **AH S133aL/P145S** | 1.152E-9 | 1/7726.0 |  | No binding | ^#^ |
| **AH T144K/P145S** | 3.050E-8 | 1/292.0 |  | 2.767E-8 | 74.0 |
| **AH S133aL/T144K/P145S** | 4.812E-11 | 1/184954.0 |  | No binding | ^#^ |

NOTE. KD(M) The binding affinity of FLD21.140 and AVFluIgG03 to the HA globular head (D55-E271) measured by bio-layer interferometry (BLI).

* From no binding to binding.

^#^ From binding to no binding.

**Table S6. Summary of HCDR3 and HCDR2 sequences and binding mode of RBS-targeting antibodies.**

| **Neutralization** | **Antibody** | **HCDR3 sequence** | **Binding mode** |
| --- | --- | --- | --- |
| H5 | FLD21.140 | ARAENLLS**P**YLAEGFDP | Hydrophobic loop-insertion binding |
| H5 | AVFluIgG03 | VRDDS**Y**DGGGHYGLHNWFDS | Hydrophobic loop-insertion binding |
| H5 | H5.3 | ARVALF**D**ILTGGWFDP | Mimic receptor binding |
| H1 | 5J8 | ARHVRSGYP**D**TAYYFDK | Mimic receptor binding |
| H1 | 1F1 | ARELLMDY**Y**DHIGYSPGPT | Hydrophobic loop-insertion binding |
| H1 | 641 I-9 | ARRRSDFETV**D**FIYHYMDV | Mimic receptor binding |
| H1 | CH65 | ARGGLEPRSV**D**YYYYGMDV | Mimic receptor binding |
| H1/H2/H3/H9 | C05 | AKHMSMQQVVSAG**W**ERADLVGDAFDV | Hydrophobic loop-insertion binding |
| H1/H2/H3/H13 | S139/1 | ILPEIG**M**T (HCDR2) | Hydrophobic loop-insertion binding |
| H1/H2/H3/H5 | F045-092 | AGPSITESHYCL**D**CAAKDYYYGLDV | Mimic receptor binding |

NOTE. Amino acid D at the tip of HCDR3 that mimics the carboxylate of sialic acid, and amino acids P/Y/W/M at the tip of HCDR3 or HCDR2 that exhibit hydrophobic interactions are bolded.


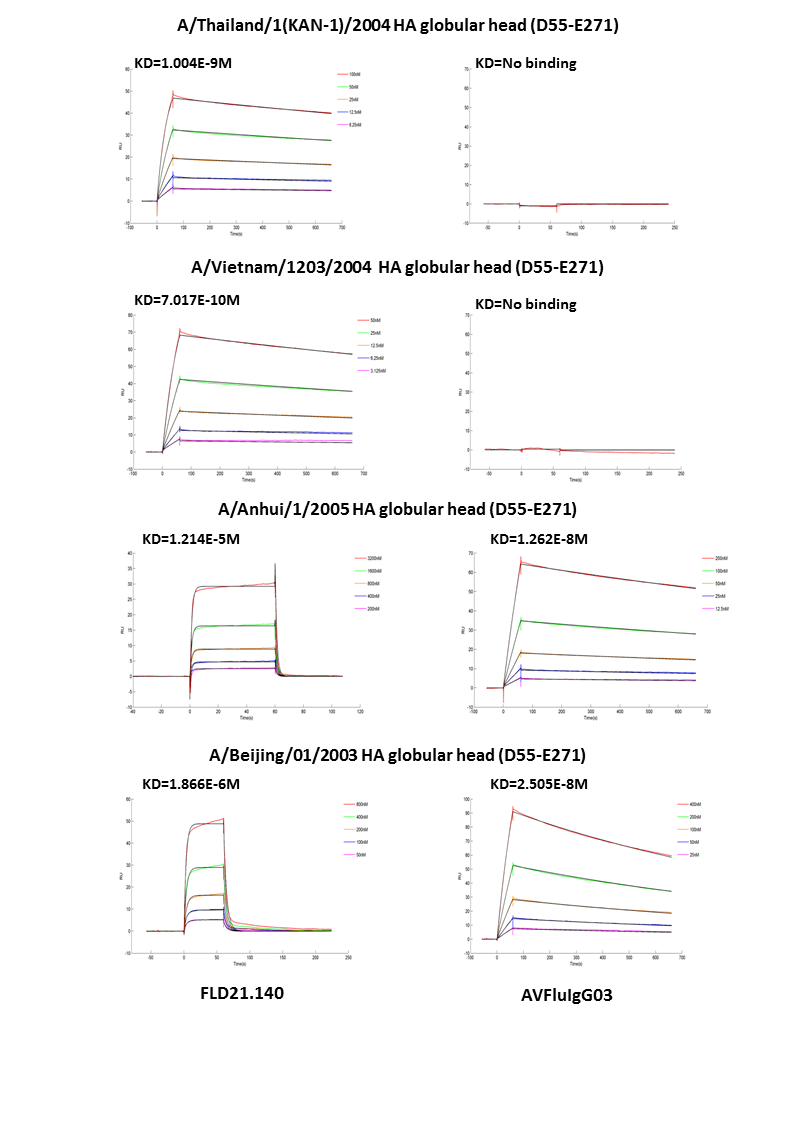


**Figure S1. Binding kinetics and affinity of FLD21.140 and AVFluIgG03 Fab to the four tested HA globular head (D55-E271) as determined by surface plasmon resonance (SPR).** The colored lines indicate the experimentally derived curves, whereas the black lines represent the fitted curves based on the experimental data.


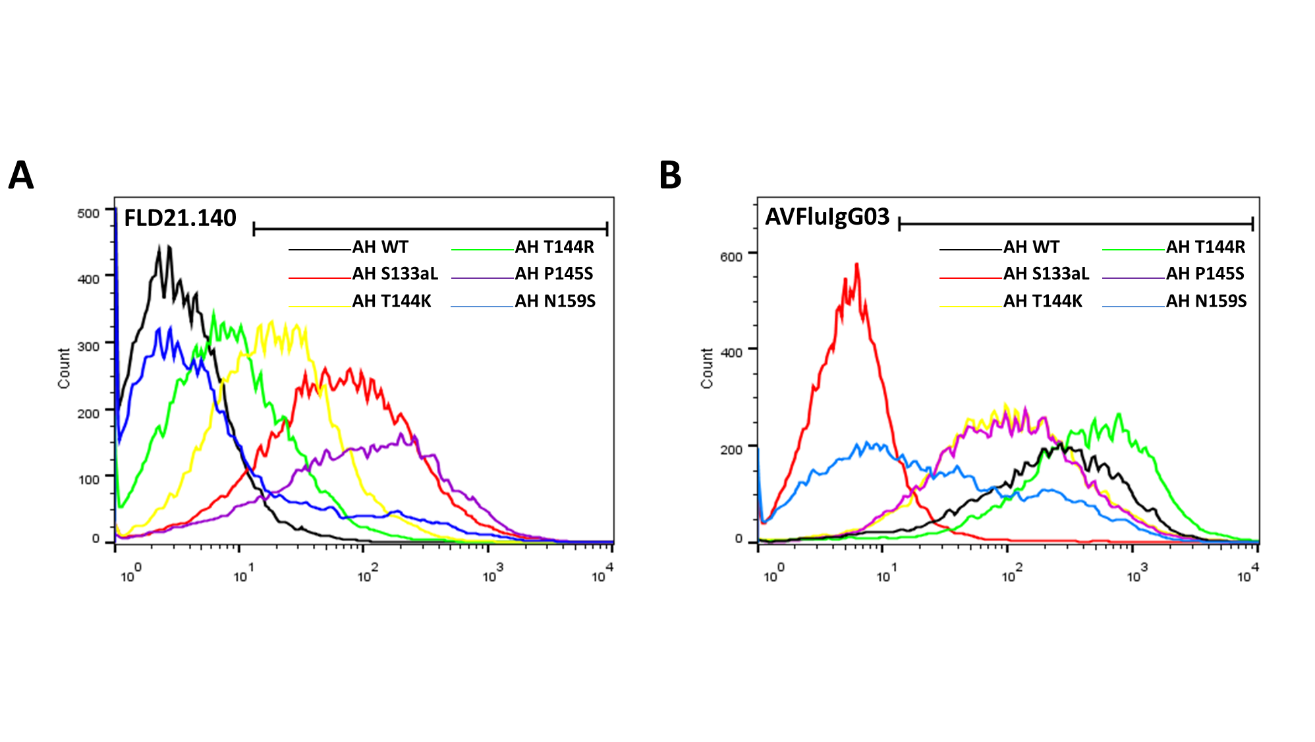


**Figure S2. Flow-cytometric analysis of FLD21.140 (A) and AVFluIgG03 (B) binding to the wild-type and single mutant HA protein of A/Anhui/1/2005 (AH for short) expressed on the cell surface of HEK293T.**


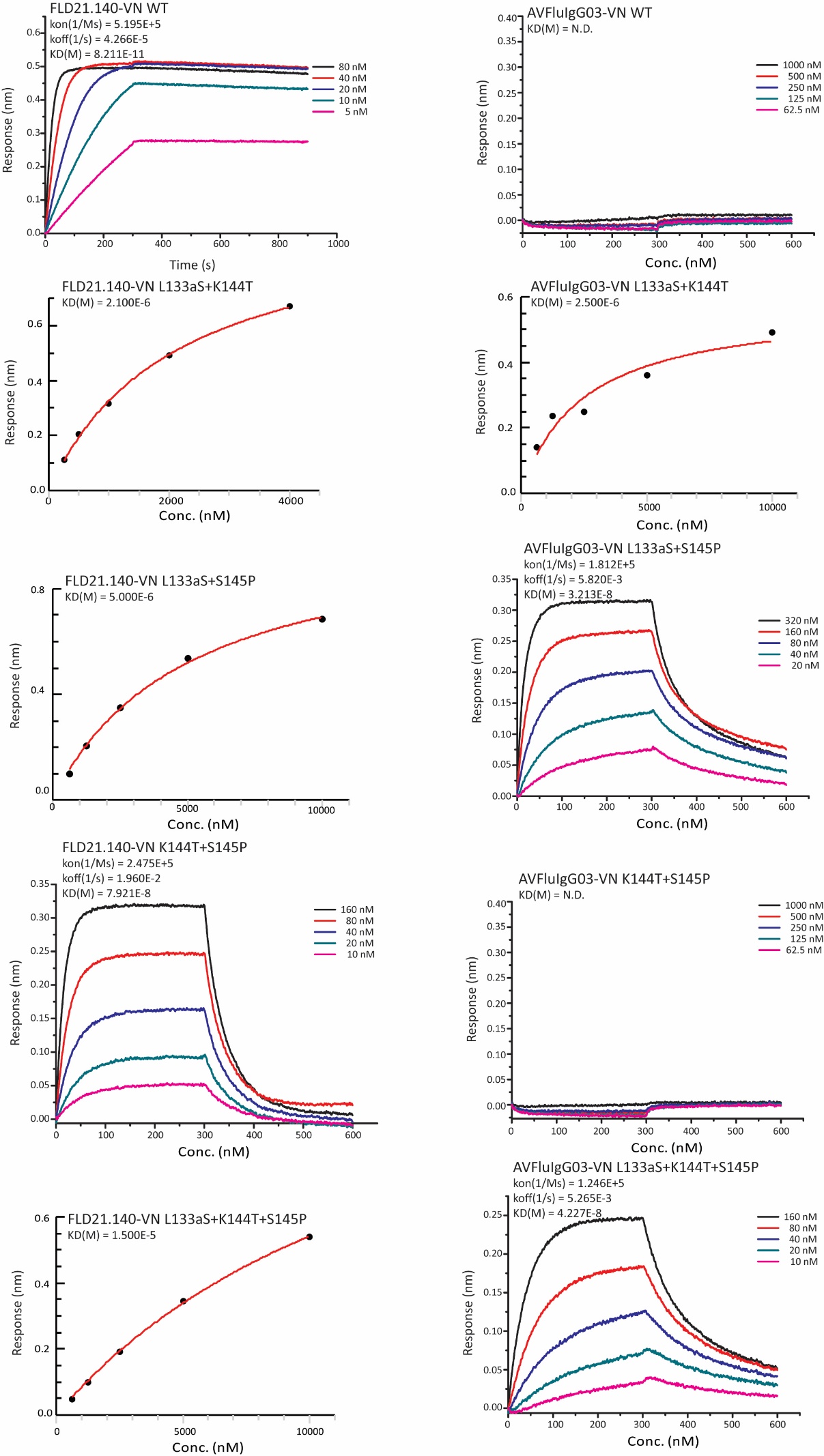

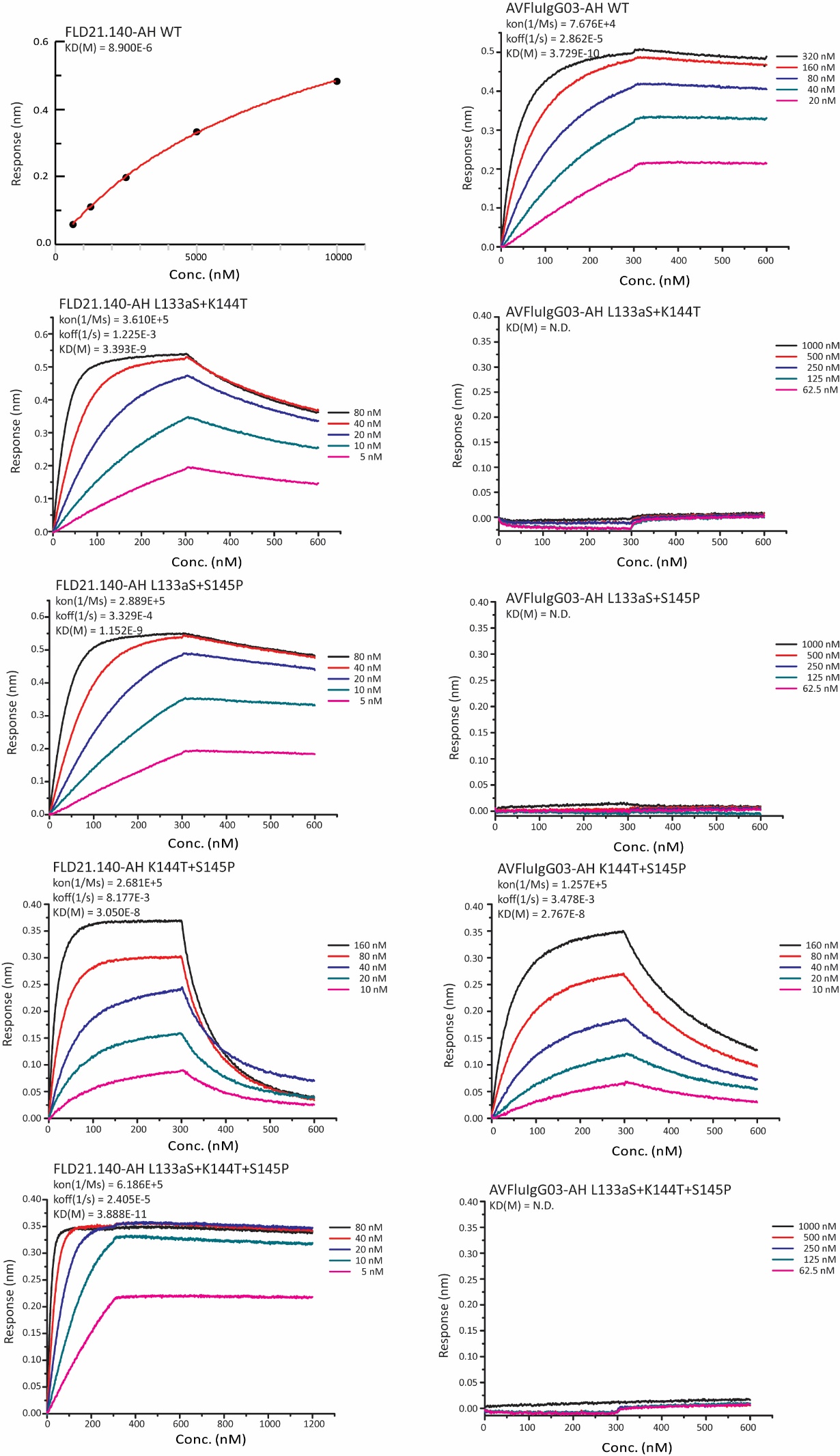


**Figure S3. Binding kinetics of FLD21.140 or AVFluIgG03 to the wild-type and mutant HA globular head (A/Anhui/1/2005 and A/Vietnam/1203/2004) determined by bio-layer interferometry.**
